# Case of Luxation of the Penis

**Published:** 1851-03

**Authors:** 


*Case of Luxation of the Penis.*—This curious case occurred in a lad æt. six, who fell from a cart. He was treated for the resulting contusions for some days, when it was observed that urine passed through a wound near the left buttock, and he was sent to the St. Louis. M. Nelaton on taking hold of the penis to pass a catheter, was astonished to find it quite destitute of substance, giving him the same sensation as the skin of a silkworm after the insect had quitted it,—a mere cutaneous tube bent upon itself. Feeling for the missing penis, he found a firm resistant body in the scrotum, which he recognised as the corpus cavernosum and glans, all the essential parts of the organ having thus remained in the scrotum for nine days. He introduced Sir A. Cooper's instrument for tying deeply seated arteries, through the cutaneous tube, and conducting the tube under the *corpus cavernosum,* seized this crosswise, and by a to-and-fro movement succeeded in replacing the organ. An incision was made *in perineo* to facilitate the evacuation of the urinary deposit which had occurred from the rupture of the urethra; and at the time of the report the child had not yet passed any urine by the natural channel.

As arising in a somewhat similar manner with this accident, viz., through the agency of a sudden compression, a case may be mentioned that was admitted sometime since into the Hôtel Dieu, in which one of the testes was found along the *corpus cavernosum* just under the skin as far as the glans. It was very easily reduced, and at the autopsy it was found that it had not become separated from the cord.—*British and Foreign Review, Jan.* 1851, *from Gaz. des Hôp.*, No. 86.

